# Symptomatic upper-extremity deep vein thrombosis induced by reverse S-shaped distortion of a peripherally inserted central catheter due to post-craniotomy epilepsy following traumatic brain injury: a case report

**DOI:** 10.3389/fmed.2026.1798849

**Published:** 2026-04-10

**Authors:** Shanwen Chen

**Affiliations:** Department of Neurosurgery, Beijing Shunyi District Hospital, Capital Medical University, Beijing, China

**Keywords:** anticoagulation, case report, epilepsy, peripherally inserted central catheter, traumatic brain injury, upper-extremity deep vein thrombosis

## Abstract

**Background:**

Upper-extremity deep vein thrombosis (UEDVT) is not uncommon in neurocritical patients with indwelling peripherally inserted central catheters (PICCs). Pharmacological anticoagulation following UEDVT poses a clinical challenge for these patients. Here, we present a rare mechanism contributing to UEDVT: distortion and deformation of a PICC line. A stepwise anticoagulation strategy was implemented while the PICC remained *in situ*.

**Case report:**

A 79-year-old man underwent an urgent craniotomy to remove a massive traumatic subdural hematoma. A 4 Fr single-lumen PICC was inserted after surgery via the right basilic vein using ultrasound guidance. The catheter tip was correctly positioned, and the PICC line remained stable for the 1 week. In the 2 week, the patient experienced recurrent seizures. Subsequently, progressive swelling of the right upper limb developed, attributed to the formation of right UEDVT due to a reverse S-shaped distortion of the proximal PICC, which further induced systemic hypoalbuminemia and generalized gravity-dependent pitting edema. The PICC was patent and left in place. A cautious anticoagulation strategy was adopted, starting with nadroparin calcium at 4,100 IU once daily, then twice daily, and later switched to enoxaparin at 6,000 IU twice daily. Upon recanalization of the thrombus, the regimen was sequentially transitioned to rivaroxaban 20 mg once daily and maintained until discharge. No intracranial bleeding was observed during the anticoagulation therapy.

**Conclusions:**

Epilepsy after traumatic brain injury can cause unexpected PICC distortion, markedly increasing UEDVT risk. If the catheter is still required and keeps patent, maintaining it *in situ* with carefully escalated anticoagulation may be a safe management strategy.

## 1 Introduction

Peripherally inserted central catheters (PICCs) are widely used in clinical settings, such as chemotherapy for cancer patients, medication administration for critically ill patients, and neonatal care. Compared with central venous catheters, the primary advantage of PICCs lies in their suitability for long-term indwelling. However, prolonged PICC retention is associated with several complications, including upper-extremity deep vein thrombosis (UEDVT), catheter-related bloodstream infections, and mechanical issues, with an overall incidence rate ranging from 9.5 to 38.6% (1). Among these, UEDVT represents the most prevalent complication following PICC placement (1, 2). Anticoagulation therapy for UEDVT poses a potential risk of bleeding, with reported rates of major bleeding ranging from 2.1 to 28.6% (2–4).

Patients with severe traumatic brain injury (TBI) often require long-term intravenous therapy, including parenteral nutrition, hyperosmolar diuretics for reducing intracranial pressure, vasoactive agents for regulating blood pressure, nimodipine for managing vasospasm, and antimicrobial agents for controlling infectious complications. These medications often cause significant irritation, making peripheral veins unsuitable for prolonged administration. Thus, PICCs emerge as a viable alternative. In various neurocritical care settings, the incidence of PICC-associated UEDVT has been reported to range from 5.5 to 8.4% (5–7). For neurocritical patients, anticoagulation therapy following UEDVT poses a clinical challenge due to the elevated risk of intracranial hemorrhage. Moreover, there are no established authoritative guidelines on whether to retain or remove the PICC line after thrombosis.

Herein, we present a case of severe TBI characterized by an uncommon proximal PICC deformation that formed a reverse S-shaped kink, which triggered the development of UEDVT. Through careful yet effective anticoagulation management while retaining the PICC line *in situ*, the thrombus was successfully recanalized, and the distorted catheter spontaneously reverted to its normal configuration.

## 2 Case presentation

**Figure 1:**
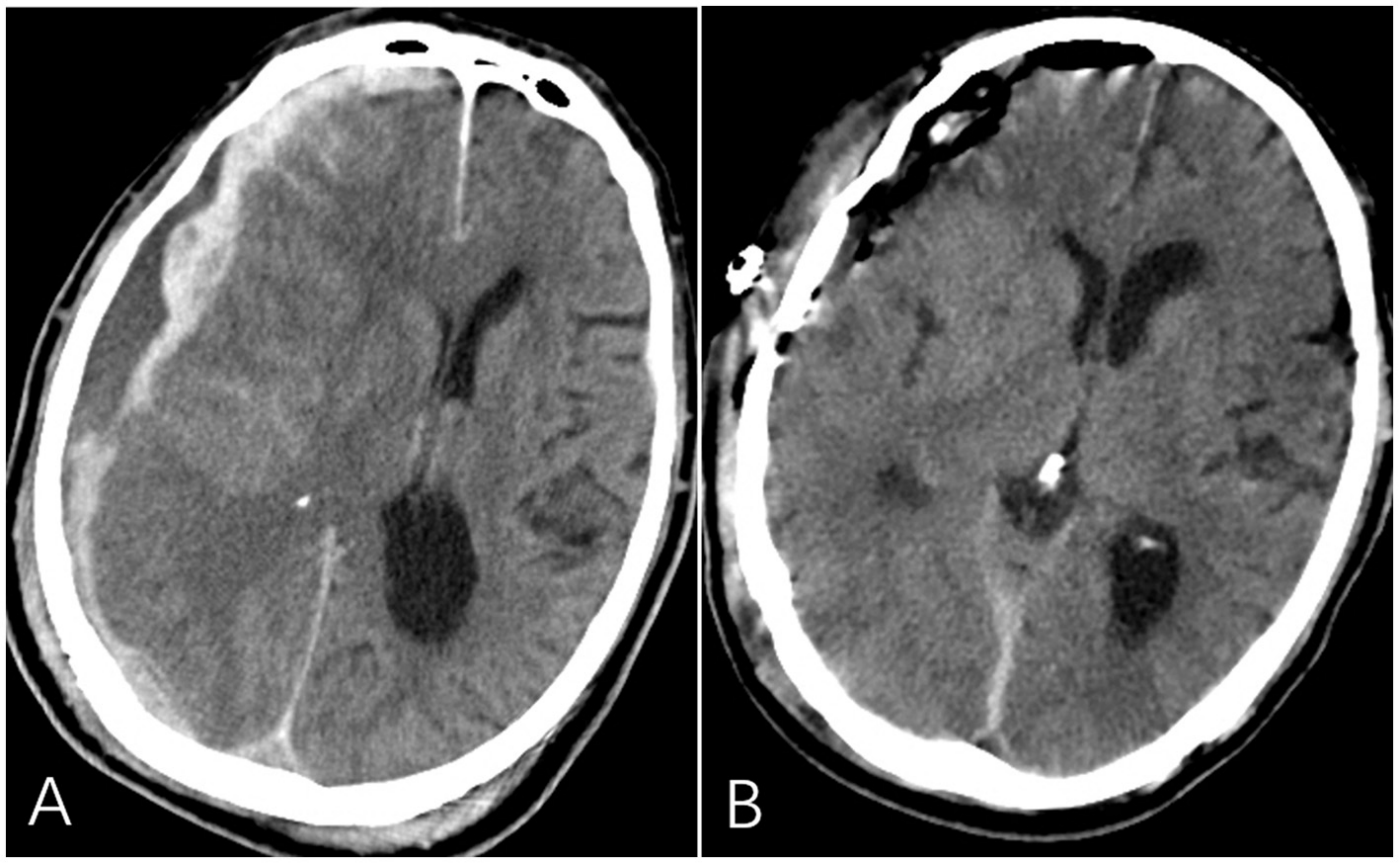
Pre- and post-operative findings on head computed tomography (CT) scans. **(A)** A massive traumatic subdural hematoma measuring 20 mm in thickness on the right frontotemporoparietal regions was shown on the preoperative head CT scan. **(B)** The subdural hematoma was completely removed through an emergency craniotomy.

A 79-year-old man was admitted to our department on March 21, 2025, due to loss of consciousness for 1.5 h after a car accident. He had a history of hypertension and diabetes. On admission, the patient was in a comatose state with a Glasgow Coma Scale score of 4. The pupil diameters were 2.5 mm on the left and 3.5 mm on the right, with no light response. No subcutaneous edema was observed in the trunk and extremities. An emergency head computed tomography (CT) scan showed a right frontotemporoparietal subdural hematoma measuring 20 mm in thickness and a midline shift of 13 mm (Figure 1A). Laboratory examinations showed a white blood cell count of 10.13 × 10^9^/L, red blood cell count of 5.40 × 10^12^/L, hemoglobin level of 166 g/L, and platelet count of 230 × 10^9^/L. Coagulation tests indicated a prothrombin time (PT) of 11.0 s, an activated partial thromboplastin time (APTT) of 24.3 s, an international normalized ratio (INR) of 1.00, a fibrinogen level of 3.52 g/L, and a D-dimer level of 16.49 mg/L. The patient was diagnosed with an acute traumatic subdural hematoma and traumatic brain herniation.

**Figure 2:**
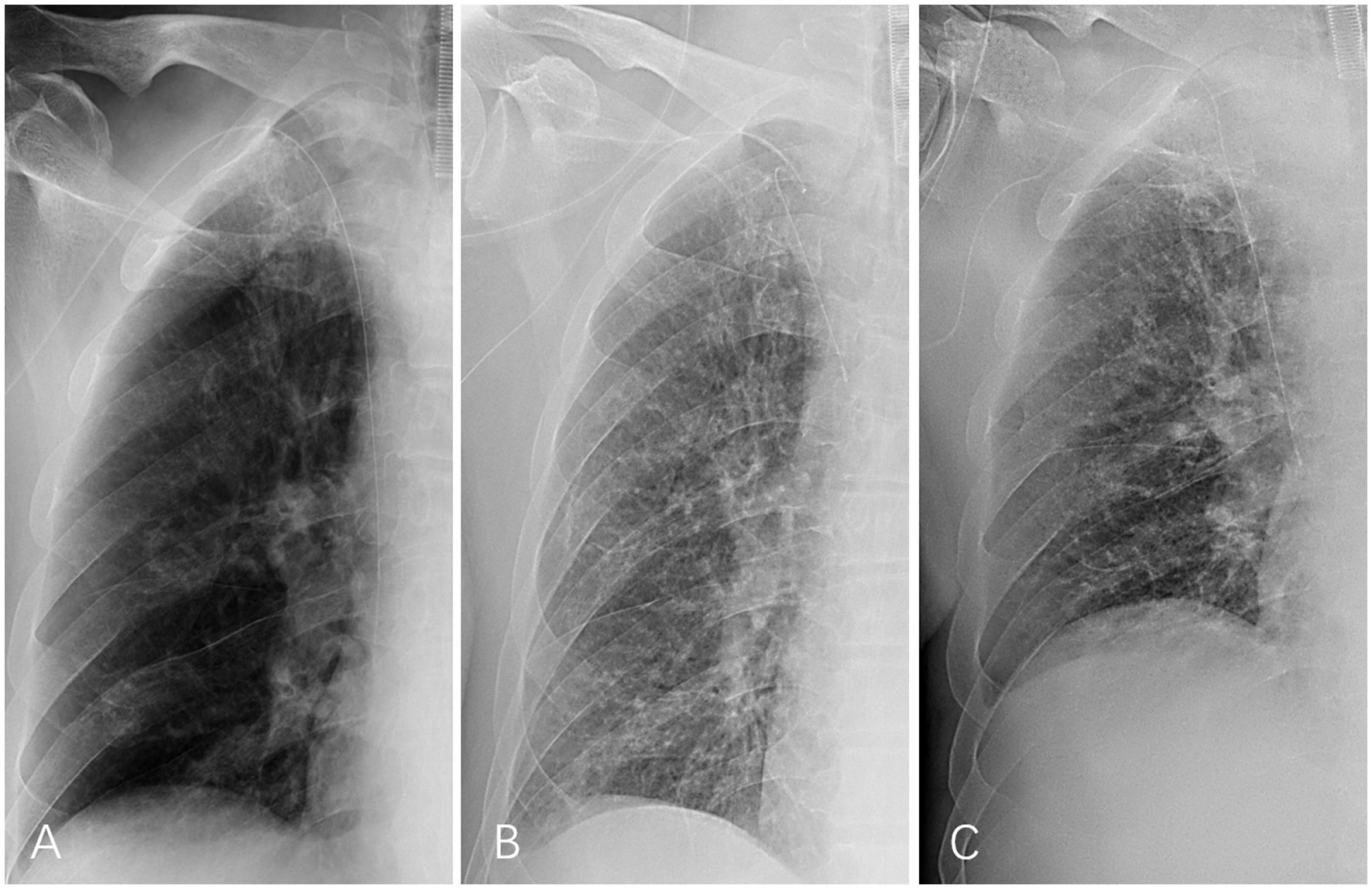
Serial chest X-ray findings during the therapy. **(A)** The PICC line was placed and ran straight along the vessel, with the tip correctly positioned at the junction of the superior vena cava and right atrium. **(B)** The proximal PICC segment remained tortuous on the chest X-ray obtained on April 12. **(C)** The proximal PICC segment returned to its normal shape on April 18.

An urgent right frontotemporoparietal craniotomy was performed to evacuate the subdural hematoma. Intraoperative findings revealed multiple cortical contusions and lacerations, along with minor hematomas in the frontal cortex, all of which were addressed simultaneously (Figure 1B). Postoperatively, the patient was transferred to the neurointensive care unit for comprehensive management. Due to difficulties with peripheral venous access, a PICC was placed in the afternoon of the surgery. Ultrasound examination confirmed the absence of thrombosis in the bilateral upper extremity veins, and a 4 Fr single-lumen PICC was inserted via the right basilic vein under ultrasound guidance using the modified Seldinger technique. The catheter tip was correctly positioned at the junction of the superior vena cava and right atrium, as verified by a bedside chest X-ray (Figure 2A). In light of the patient's clinical condition, supportive therapies were initiated, including mechanical ventilation, sedation and analgesia, and parenteral nutrition, along with nimodipine to prevent cerebral vasospasm. A follow-up head CT scan demonstrated complete evacuation of the subdural hematoma with no evidence of delayed hemorrhage. With ongoing treatment, his condition gradually improved, as evidenced by eye opening in response to painful stimuli.

**Figure 3:**
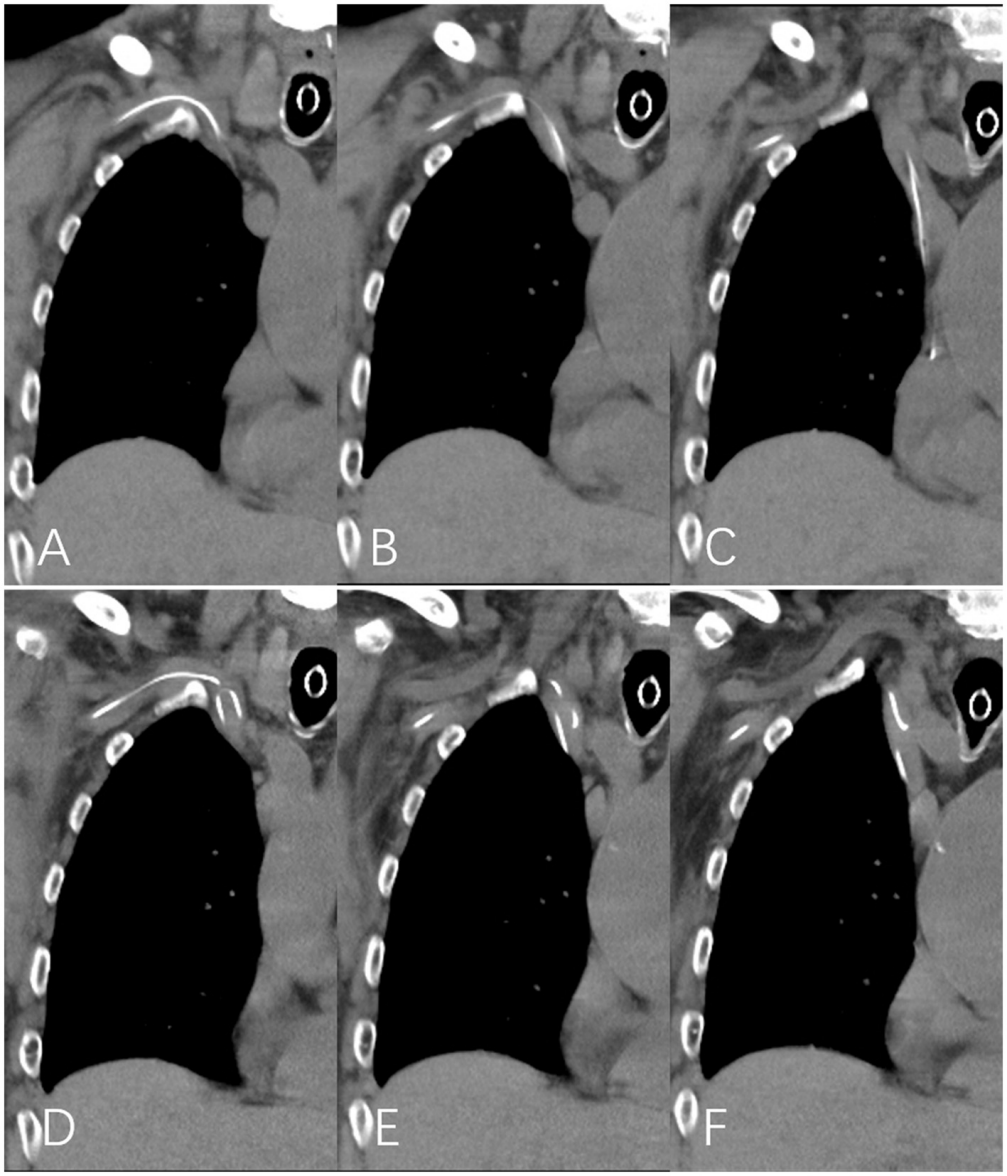
The catheter appearances on chest CT scans. **(A–C)** The PICC line tip position and shape were confirmed to be normal on March 28. **(D–F)** The proximal PICC segment formed a reverse S-shaped kink in the right brachiocephalic vein on April 2, with the tip displaced to the upper third of the superior vena cava.

However, between March 27 and April 5, the patient experienced recurrent epileptic seizures, involving the left limb and face, with episodes lasting up to approximately 1 min. The seizures were gradually managed by administering intravenous sodium valproate, nasogastric levetiracetam, and intramuscular phenobarbital. The PICC line was verified to retain its initial configuration by a chest CT scan conducted on March 28 (Figures 3A–C). On April 2, mild swelling developed in the right dorsal hand. On the same day, a repeat chest CT scan was obtained. Unfortunately, the mediastinal window was overlooked, which revealed that the proximal PICC segment had formed a reverse S-shaped kink in the right brachiocephalic vein, with the tip displaced to the upper third of the superior vena cava (Figures 3D–F). By April 3, the swelling had extended to encompass the entire right upper limb. A Doppler ultrasound examination showed widened lumens of the right axillary, brachial, radial, and ulnar veins, filled with hypoechoic material and exhibiting no detectable blood flow, indicating right UEDVT. The PICC lumen was confirmed patent by flushing with heparinized saline. On that day, the complete blood count indicated a platelet count of 311 × 10^9^/L, and the coagulation profile revealed a PT of 12.4 s, an APTT of 27.1 s, an INR of 1.08, and a D-dimer level of 4.05 mg/L.

**Figure 4:**
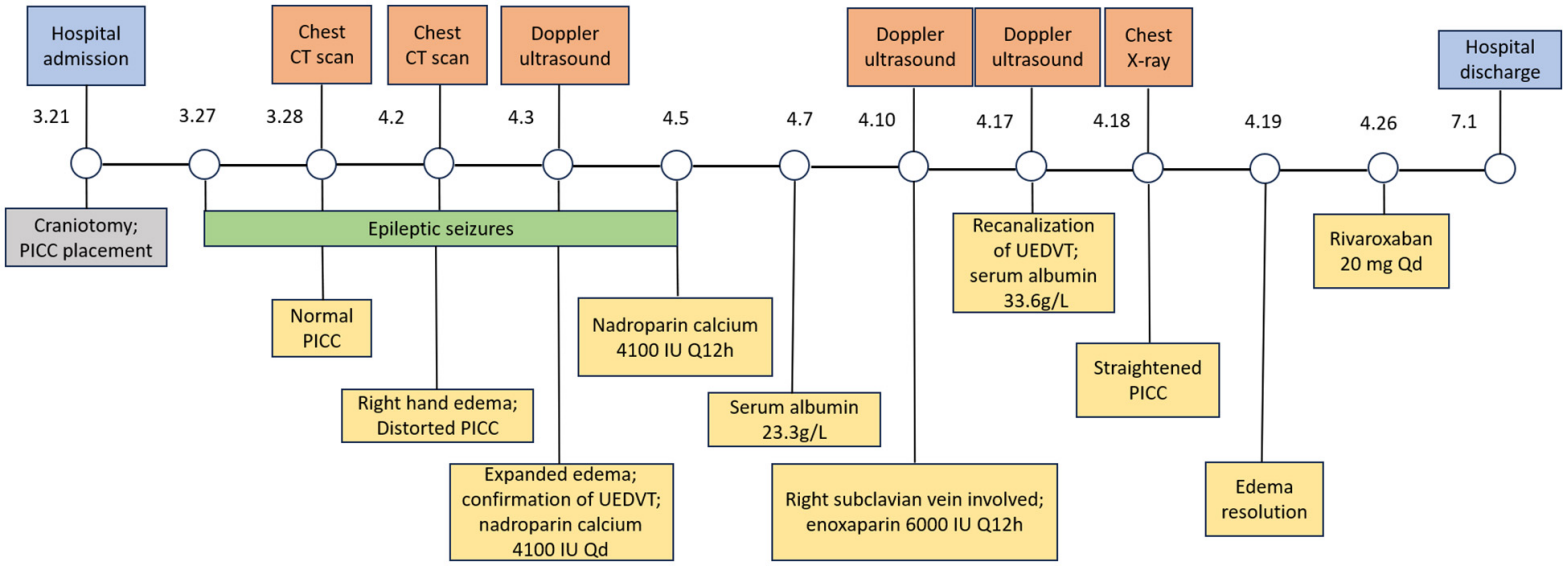
Clinical timeline of the patient's diagnostic and therapeutic course. The timeline summarizes symptom onset and progression, radiologic and ultrasonic evaluations, stepwise anticoagulation, and interventional outcome.

Considering the risk of intracranial hemorrhage 13 days post-operation, a stepwise antithrombotic therapy was initiated with subcutaneous nadroparin calcium, initially at 4,100 IU once daily and increased to every 12 h after 2 days. However, the patient experienced progressive swelling in the right upper limb, accompanied by generalized gravity-dependent pitting edema, involving the left hand, back, buttocks, posterior thighs, and scrotum. Serum albumin levels fell from 27.3 g/L on April 4 to 23.3 g/L on April 7, prompting intravenous administration of 10 g of human albumin daily to address hypoalbuminemia. A follow-up ultrasound conducted on April 10 revealed the thrombus extending from the right axillary to subclavian veins, with partial flow signals detected in the brachial, radial, and ulnar veins, as well as new calf muscle vein thrombosis in the right lower limb. The proximal PICC still appeared tortuous on the chest X-ray obtained on April 12 (Figure 2B). Due to progression of thrombosis and an evaluated body weight of 65 Kg, anticoagulation therapy was transitioned to enoxaparin 6,000 IU every 12 h. Despite these interventions, limb, and trunk edema persisted, and serum albumin levels remained around 23 g/L; consequently, human albumin was increased to 10 g twice daily. Finally, these ongoing adjustments to the therapeutic regimen yielded promising outcomes. On April 17, a repeat ultrasound showed resolution of the right UEDVT with restored venous patency, and the serum albumin level increased to 33.6 g/L. The proximal PICC segment straightened and returned to its normal configuration, as confirmed by a follow-up chest X-ray on April 18 (Figure 2C). By April 19, edema in the right upper limb had subsided entirely, followed by a gradual resolution of generalized subcutaneous edema. During this period, the PICC lumen was flushed daily with heparinized saline to maintain patency, and fluid administration was resumed after thrombus resolution was confirmed. Enoxaparin was administered until April 25, 3 weeks after thrombus detection, followed by sequential therapy with rivaroxaban 20 mg once daily. The patient did not experience any further venous thromboembolic events until discharge on July 1, at which point the PICC line was removed. The overall clinical course and key diagnostic and therapeutic milestones are summarized in a clinical timeline (Figure 4).

During anticoagulation therapy, a head CT scan revealed no new intracranial hemorrhage. On April 8, pink macroscopic hematuria was observed in the urine collection bag, which resolved after bladder irrigation. Subsequent multiple urinalyses showed up to 15–20 red blood cells per high-power field on microscopy. The stool occult blood test was negative. No significant gastrointestinal or mucocutaneous bleeding was observed.

## 3 Discussion

In this case report, we describe a novel mechanism of PICC-related UEDVT: distortion of the proximal catheter into a reverse S-shaped configuration, leading to local hemodynamic disturbance. To our knowledge, this is the first documented instance of such a mechanism implicated in the development of UEDVT.

Although the placement of a PICC line offers considerable convenience for treatment, its potential complications should not be overlooked. Among these complications, catheter-related UEDVT is the most prevalent noninfectious issue following PICC insertion, accounting for approximately 5%−10% of all deep vein thrombosis (DVT) cases (2). A systematic review by Chopra et al. in 2013 indicated that the incidence of PICC-related DVT in critically ill patients could reach 13.91% (8). Furthermore, a retrospective analysis of 6,607 trauma patients at a Level I trauma center reported a UEDVT incidence of 14.6% (9). In recent years, the establishment of specialized PICC care teams and the optimization of clinical practices have led to a significant decline in PICC-related UEDVT. In 2025, Li et al. conducted a retrospective study of neurocritical care patients and reported an incidence of PICC-related large vein thrombosis of 0.5% (10).

As is known, patients with severe trauma often exhibit a hypercoagulable state, which is attributed to intrinsic alterations in blood properties and vascular endothelial injury. This condition, further exacerbated by diminished muscle pump function in the extremities due to limited mobility, markedly increases the risk of DVT in the limbs (11). These factors lay the foundation for the occurrence of UEDVT, on which PICC, as a foreign object in the blood vessel, significantly increases the risk of UEDVT. A crucial preventive measure for UEDVT is ensuring accurate PICC tip positioning (12). According to the practice standards set by the Infusion Nurses Society (13, 14), the catheter tip should be placed in the lower third of the superior vena cava or near the cavoatrial junction. At our institution, PICC insertion is performed under ultrasound guidance using the modified Seldinger technique, followed by a bedside chest X-ray to verify correct tip placement. In cases of malposition, prompt adjustments are made. These procedures represent standard clinical practices. Additionally, catheter diameter has been identified as a factor associated with UEDVT, with numerous studies demonstrating a significant positive correlation between the two. A meta-analysis conducted by Schears et al. in 2021 showed that the overall risk of DVT following PICC placement was 8.9%. Subgroup analysis by catheter diameter indicated that the risk for smaller-diameter catheters (3 Fr and 4 Fr) was 2.3%. In contrast, for larger-diameter catheters (5 Fr and 6 Fr), it was 12.7%, representing an increase of over 10% (15). A subsequent meta-analysis by Bahl et al. in 2022 showed DVT rates was 5.08% in intensive care unit (ICU) patients and 4.58% in non-ICU patients, with no statistically significant difference; and the DVT incidence rates for 3 Fr, 4 Fr, 5 Fr, and 6 Fr PICCs were 0.89%, 3.26%, 5.46%, and 10.66%, respectively (16). Liem et al. identified a strong correlation between PICC diameter and thrombosis risk, with thrombosis rates of 1% for 4 Fr, 6.6% for 5 Fr, and 9.8% for 6 Fr catheters, while no association was observed with 3 Fr PICCs (17). It has become a consensus recommendation to select a catheter with the smallest external diameter and the fewest lumens that adequately meets patient needs (12–14). Accordingly, to minimize the risk of DVT, we consistently use 3 Fr or 4 Fr single-lumen catheters.

However, the use of smaller catheters is associated with an increased risk of mechanical complications, such as catheter occlusion, displacement, and malposition (12). The reduced lumen diameter predisposes them to occlusion, while their relatively flexible structure allows the catheter tip to migrate distally in response to elevated intrathoracic pressure transmitted to the mediastinum. In severe cases, this may result in displacement into the internal jugular vein. In the present case, the patient remained intubated postoperatively and required frequent sputum suction, which induced passive coughing and, consequently, abrupt increases in intrathoracic pressure. However, because endotracheal suction was initiated early in the postoperative period and the right UEDVT developed more than 1 week post-surgery (between March 28 and April 2), the temporal correlation between the two events appears weak. During this interval, the patient received enteral nutrition and medications via a nasogastric tube, without experiencing significant vomiting or hiccups. Conversely, the onset of recurrent seizures after March 27 closely coincided with the timeframe of UEDVT formation. During convulsive episodes, sustained elevations in intrathoracic pressure reached a critical level that was sufficient to cause migration of the PICC tip toward the distal superior vena cava, yet insufficient to dislodge the entire terminal segment into the brachiocephalic or subclavian vein. As a result, a reverse S-shaped kink formed, increasing the catheter's cross-sectional area and disrupting local blood flow, directly leading to UEDVT. The ensuing UEDVT impeded venous return in the right upper limb, causing transudative edema, and a subsequent progressive decline in serum albumin levels, which, in turn, induced extensive subcutaneous edema. Fortunately, the catheter remained patent throughout this period and effectively resumed venous access following thrombus recanalization.

Therefore, prophylactic antithrombotic therapy is deemed a reasonable intervention. However, there remains a persistent and unresolved conflict between the benefits of pharmacological thromboprophylaxis and the concomitant risk of bleeding. A retrospective cohort study conducted by Walusimbi et al. in 2022, including 381 trauma patients, identified PICC placement and TBI as independent risk factors for UEDVT (9). The incidence of UEDVT was 7.5% among patients receiving prophylactic low-molecular-weight heparin, compared with 21.5% among those without prophylaxis, indicating a statistically significant difference (9). Nevertheless, the study did not assess the bleeding risk associated with prophylactic anticoagulation. A Cochrane meta-analysis in 2013 demonstrated that pharmacological prophylaxis was more effective than mechanical methods in reducing the risk of DVT in trauma patients, but also increased the risk of bleeding with a risk ratio of 2.04 (11). Consequently, current guidelines recommend mechanical thromboprophylaxis over pharmacological methods in critically ill patients who are actively bleeding or at a high risk for hemorrhage (18, 19). In our clinical practice, given the potential risk of intracranial bleeding in patients with severe TBI, we abstain from pharmacological prophylaxis, even in cases where long-term PICC indwelling is required. Instead, we conduct ultrasound surveillance every 10 to 14 days to monitor venous patency.

Current guidelines offer recommendations of varying strength regarding catheter removal and anticoagulation strategies following PICC-related UEDVT. The American College of Chest Physicians (CHEST) evidence-based guidelines on antithrombotic therapy for venous thromboembolism recommend against catheter removal in patients with catheter-associated UEDVT, provided the catheter remains functional and clinically necessary (weak recommendation, low-quality evidence) (18). Additionally, anticoagulation therapy should be maintained as long as the catheter remains in place (strong recommendation, moderate-quality evidence) (18). A retrospective study from China, focusing on elderly patients with PICC-related UEDVT, indicated that both catheter removal and antithrombotic therapy facilitated thrombus resolution. However, re-catheterization at a new site was still associated with recurrent catheter-related thrombosis (20). Therefore, catheter retention is recommended when it remains essential for therapy and shows no signs of infection. In this instance, a prudent clinical decision was made. Following the diagnosis of UEDVT, the PICC lumen was confirmed patent. Given the patient's requirement for prolonged intravenous therapy, the catheter was retained. Daily flushes with heparinized saline were administered. Concurrently, the patient was prescribed low-molecular-weight heparin and an oral anticoagulant, with dosages adjusted based on clinical monitoring. Thrombus resolution was achieved within 2 weeks, along with correction of catheter twisting and restoration of the catheter's normal configuration. During the anticoagulation therapy, only one minor episode of macroscopic hematuria was observed, with no major bleeding events or new intracranial hemorrhages. These findings suggest that pharmacological anticoagulation during the subacute phase of TBI may be safe.

The patient's clinical management had a few shortcomings. Initially, the dorsal hand edema was insufficiently addressed, as it was hastily attributed to gravitational dependency. Additionally, the chest CT scan performed on the same day was not thoroughly reviewed. Although an earlier diagnosis of UEDVT might not have substantially impacted the clinical course in this case, this instance underscores the imperative of meticulous evaluation of limb swelling in patients with PICC indwelling. It is recommended that regular radiographic monitoring of catheter position and configuration be integrated into standardized protocols. Lastly, anti-Xa activity monitoring was not performed after low-molecular-weight heparin administration due to facility limitations at our institution.

In summary, epilepsy after TBI can mechanically deform a PICC via seizure-induced intrathoracic pressure surges, creating a kink and significantly increasing UEDVT risk. Given this, regular radiographic monitoring of catheter morphology and vigilance for early signs of UEDVT are essential in neurocritical care. When the PICC is still needed and remains patent, leaving it *in situ* with carefully escalated anticoagulation can be safe, even in subacute TBI.

## Data Availability

The raw data supporting the conclusions of this article will be made available by the authors, without undue reservation.
