# Supplementary figures and images for: Symptomatic upper-extremity deep vein thrombosis induced by reverse S-shaped distortion of a peripherally inserted central catheter due to post-craniotomy epilepsy following traumatic brain injury: a case report
Source: Front Med (Lausanne). 2026 Apr 10;13:1798849. doi: 10.3389/fmed.2026.1798849 (PMC13106069; doi:10.3389/fmed.2026.1798849)

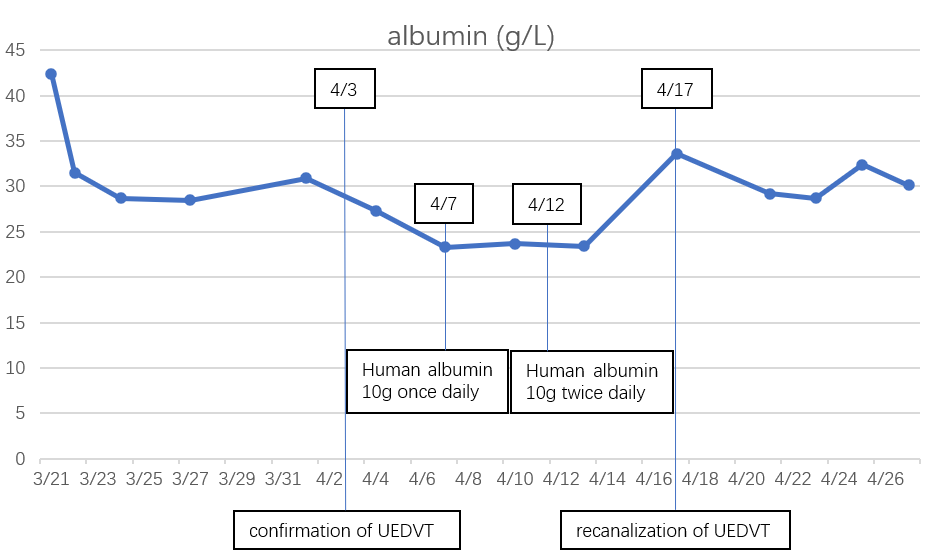

Supplement: Supplementary file 1 [file Image_1.TIF]
